# Association between acrylamide exposure and the odds of developmental disabilities in children: A cross-sectional study

**DOI:** 10.3389/fpubh.2022.972368

**Published:** 2022-09-30

**Authors:** Fanchao Meng, Yanjie Qi, Yuanzhen Wu, Fan He

**Affiliations:** The National Clinical Research Center for Mental Disorder and Beijing Key Laboratory of Mental Disorders, Beijing Anding Hospital, Capital Medical University, Beijing, China

**Keywords:** acrylamide, developmental disabilities, children, restricted cubic splines (RCS), NHANES

## Abstract

**Background:**

The association between acrylamide exposure and the odds of developmental disabilities (DDs) is unclear. We conducted this analysis to explore whether acrylamide exposure is related to DDs.

**Methods:**

We analyzed a sample of 1,140 children aged 6–17 years old from the US National Health and Nutrition Examination Survey 2013–2014 to 2015–2016. DDs were determined by reports of parents. Acrylamide exposure was evaluated by the hemoglobin adducts of acrylamide (HbAA) and its major metabolite glycidamide (HbGA). We investigated the association using binomial logistic regression analysis by taking HbAA and HbGA as continuous or quartile variables. Restricted cubic splines (RCS) were used to explore the non-linear relationship between HbAA or HbGA and the odds of DDs. Interaction analysis and propensity score matching (PSM) were used to validate the results.

**Results:**

A total of 134 participants were reported to have DDs. The median level of HbAA and HbGA was 41.6 and 40.5 pmol/g Hb, respectively. HbAA and HbGA were not associated with the odds of DDs when taken as continuous variables. When divided into quartiles, there was no evidence for a linear trend for HbAA and HbGA. RCS showed that there was a J-shaped association between HbGA and the odds of DDs (*P* for non-linearity, 0.023). The results were consistent in interaction analysis by age, gender, and race, and after PSM.

**Conclusion:**

HbGA level was associated with the odds of DDs in a J-shaped manner among children. Further investigation is warranted to determine the causality and underlying mechanisms.

## Introduction

Developmental disabilities (DDs) are a group of lifelong conditions due to various degrees of impairment in physical, learning, language, or behavior areas. These conditions begin during the developmental period, may impact daily functioning, and usually last throughout a person's lifetime. DDs are significant public health problems both in China and around the world (1, 2). In the US, the prevalence of any DDs among children aged 3–17 years was estimated to be 16.2–17.8% and appeared to be increasing in the recent decade (3). The etiology of DDs is not fully clarified. However, exposure to environmental pollutants has been suggested to play a critical role in the development of DDs (4).

Acrylamide, an industrially produced α, β-unsaturated (conjugated) reactive molecule, is used worldwide to synthesize polyacrylamide. Food and tobacco smoke are the major sources of acrylamide exposure for the general population (5–7). It has been widely found in bakery products such as potato chips, French fries, bread, biscuits, and coffee. Exposure from other sources is significantly less than from food or tobacco (8). The effects of acrylamide in cells, tissues, animals, and humans have been extensively investigated, and it has been implicated as a possible risk factor for health from life development to senescence (9–11).

The hemoglobin (Hb) adducts of acrylamide (HbAA) and its major metabolite glycidamide (HbGA) at the N-terminal valine are well-established biomarkers of internal acrylamide exposure. They were employed to estimate the internal dose among occupational workers and the general population (12, 13). Previous studies have explored the controversial associations of HbAA and HbGA with different health conditions such as cancer, cardiovascular disease, and cognitive dysfunction (14–16). While it has been well documented in animal studies that even low exposures to environmental acrylamide during the critical periods of brain development can cause neurotoxic effects (17), only two human population studies reported the possible association between dietary acrylamide intake and neurological symptoms (18, 19). Moreover, the association between acrylamide exposure and the odds of DDs has not been reported currently.

In this study, we aimed to demonstrate whether daily acrylamide exposure was associated with the odds of DDs in children by examining the participants in a nationally representative cohort from the National Health and Nutrition Examination Survey (NHANES) collected between 2013–2014 and 2015–2016 in the United States.

## Methods

### Study population

NHANES is a cross-sectional survey that includes an in-home interview, physical examination, and laboratory tests to collect data on demographics, health, and nutritional status of the US civilian noninstitutionalized population. It uses a complex, multistage, probability sampling design to ensure nationwide population representativeness. NHANES is conducted by the National Center for Health Statistics (NCHS) at the Centers for Disease Control and Prevention, and it has been approved by the NCHS Ethics Review Board. The data are released in 2-year cycles and available online for public access. Written informed consent was obtained from all participants.

We used data of participants aged 6–17 years old from NHANES between 2013–2014 and 2015–2016, which were the NHANES cycles that included both information on DDs and acrylamide exposure measurements.

### Measurement of HbAA and HbGA

Whole blood samples were collected during the physical examination, stored frozen (−20°C), and sent to the central laboratory for the measurement of HbAA and HbGA. Only one-third of interviewed participants aged 6 years and older were sampled for HbAA and HbGA measurement. The measurement is based on the modified Edman reaction, which uses the effect of N-alkylated amino acids being able to form Edman products in neutral or alkaline conditions without changing the pH to acidic conditions required in conventional Edman reaction procedures. The experimental details have been described in the Laboratory Method Files of NHANES (20). The methods were consistent for NHANES 2013–2014 and 2015–2016. The detection limits for the levels of HbAA and HbGA were 3.90 and 4.90 pmol/g Hb.

### Outcome ascertainment and covariables

DDs were defined as a positive answer to the question “Does your child receive Special Education or Early Intervention Services?”. Only parents of children aged 3–17 years old were asked.

Covariables were identified based on the literature on DDs and environmental pollutants among children (21–23). Included covariables were age, gender, race, family income, body mass index (BMI), normal birth weight (>5.5 lb), maternal smoking during pregnancy, health insurance coverage, and the number of annual health care visits. Race/ethnicity was categorized as Hispanic, non-Hispanic white, non-Hispanic black, and others. The family income was measured by the income-to-poverty ratio, which was defined as annual family income divided by the poverty threshold adjusted for family size and inflation. BMI was determined as the weight in kilograms divided by height in meters squared. Current household tobacco smoke exposure was not included as it was a major source of acrylamide exposure according to the literature (7), and there was major multicollinearity (variance inflation factor >5) in the multivariable analysis if it was included.

### Statistical analysis

All statistical analyses were performed in R version 4.1.3 with the “Survey” package after accounting for the complex sampling design. HbAA and HbGA were measured in a one-third subsample of interviewed participants, and 4-year subsample weights were constructed following recommendation (24). All statistical tests were 2-sided, and *P* < 0.05 was considered statistically significant.

There were 4,834 participants aged 6–17 years old in NHANES 2013–2014 and 2015–2016. Participants without information on DDs were excluded (*N* = 8). Those without measurement of HbAA or HbGA were also excluded (*N* = 3,686). This resulted in a total of 1,140 children (134 with DDs and 1,006 without DDs) available for analysis.

The levels of HbAA and HbGA were right-skewed and natural-log transformed for the analysis. Continuous variables were expressed as means and categorical variables as percentages. The difference in continuous variables between those with and without DDs was examined with a *t*-test and the difference in categorical variables was examined with a Chi-squared test. The binomial logistic regression model was applied to evaluate the relationship between HbAA or HbGA and the odds of DDs. First, HbAA or HbGA was modeled as continuous exposure after log transformation. Second, they were divided into quartiles based on the weighted distributions and were modeled as ordinal variables with the first quartile as the reference. Third, restricted cubic splines (RCS) were used in the logistic regression model to explore the potential non-linear relationship between HbAA or HbGA and the odds of DDs. As recommended, four knots were applied in the RCS to better fit the model and avoid overfitting (25). The *P*-values for non-linear trends were obtained by Wald tests for RCS coefficients. Three models were developed for the regression analysis. In model 1, no covariables were adjusted for. In model 2, age, gender, race, and income were adjusted. In model 3, BMI, normal birth weight, maternal smoking during pregnancy, health insurance coverage, and the number of annual health care visits were further adjusted based on model 2.

According to our results, there were significant differences in gender and race/ethnicity among children with and without DDs. Furthermore, there were significant age, gender, and racial/ethnic differences in the incidence of DDs according to the literature (26–28). Therefore, to explore if the association differed in subgroups classified by age (6–12 and 13–17 years), gender (male and female), and race (whites and non-whites), interaction analysis was performed by adding an interaction term between the quartiles of HbAA or HbGA and subgroup status in the regression model.

To avoid the potential bias caused by differences in the characteristics of participants, propensity score matching (PSM) was performed. PSM was performed at a ratio of 1:3 with the nearest neighbor-based approach, using the “MatchIt” package in R. Logistic regression analysis with RCS was used to evaluate the relationship between HbAA or HbGA and the odds of DDs after PSM.

## Results

The characteristics of children are presented in Table 1. Compared to those without DDs, children with DDs were more likely to be male and non-Hispanic white, less likely to have normal birth weight, and more likely to have health insurance coverage. The median level of HbAA and HbGA was 41.6 and 40.5 pmol/g Hb, respectively (Table 2).

**Table 1:** Characteristics of children included in NHANES 2013–2016 analyses.

**Characteristic**	**With DDs (*N* = 134)**	**Without DDs (*N* = 1006)**	***P*-value**
Age, years	11.2 (0.3)	11.9 (0.2)	0.060
Male, %	69.4 (4.5)	48.6 (2.3)	< 0.001
Race/ethnicity, %			0.004
Hispanic	16.0 (3.1)	25.5 (3.8)	
Non-hispanic white	61.7 (4.6)	49.5 (4.5)	
Non-hispanic black	17.9 (3.7)	13.0 (2.2)	
Other race	4.4 (1.1)	11.9 (1.6)	
Income-to-poverty ratio ≤ 1.3, %	37.4 (5.7)	28.5 (2.4)	0.105
BMI	21.2 (0.5)	21.5 (0.2)	0.671
Birth weight >5.5 lb, %	76.6 (5.0)	88.4 (1.4)	0.013
Maternal smoking during pregnancy, %	19.3 (5.9)	11.8 (2.0)	0.152
With health insurance coverage, %	98.1 (1.0)	94.2 (1.0)	0.028
Times received healthcare ≤ 1, %	33.8 (4.8)	43.1 (1.8)	0.140

Data were expressed as means with standard error in parenthesis.

BMI, body mass index; DDs, developmental disabilities; NHANES, national health and nutrition examination survey.

**Table 2:** Odds ratio (95% CI) of developmental disabilities by quartile of HbAA and HbGA.

	**Quartile 1**	**Quartile 2**	**Quartile 3**	**Quartile 4**	***P* for trend**
HbAA (pmol/g Hb)	≤ 33.7	33.7–41.6	41.6–50.3	>50.3	
Model 1	Reference	1.21 (0.59–2.50)	**2.10 (1.18–3.77)**	1.34 (0.70–2.56)	0.116
Model 2	Reference	1.02 (0.48–2.18)	1.80 (0.94–3.45)	1.17 (0.60–2.28)	0.286
Model 3	Reference	0.91 (0.37–2.25)	1.52 (0.68–3.37)	0.91 (0.40–2.05)	0.789
HbGA (pmol/g Hb)	≤ 31.2	31.2–40.5	40.5–52.4	>52.4	
Model 1	Reference	1.49 (0.89–2.50)	0.58 (0.26–1.29)	**2.16 (1.27–3.68)**	0.065
Model 2	Reference	1.32 (0.80–2.16)	0.48 (0.22–1.03)	1.71 (0.93–3.18)	0.278
Model 3	Reference	1.00 (0.49–2.07)	**0.31 (0.14–0.72)**	1.36 (0.58–3.22)	0.783

Bold fonts mean statistically significant.

CI, confidence interval; Hb, hemoglobin; HbAA, hemoglobin adducts of acrylamide; HbGA, hemoglobin adducts of glycidamide.

No covariables were adjusted for in model 1. Age, gender, race, and income were adjusted in model 2. Age, gender, race, income, body mass index, normal birth weight, maternal smoking during pregnancy, health insurance coverage, and the number of annual health care visits were adjusted in model 3.

When modeled as a continuous variable in the regression analysis, HbAA was not significantly associated with the odds of DDs in model 1 [odds ratio (OR): 1.86; 95% confidence interval (95% CI): 0.98–3.51], model 2 (OR: 1.67; 95% CI: 0.84–3.31), and model 3 (OR: 1.68; 95% CI: 0.57–4.97). When modeled as a continuous variable, HbGA was significantly associated with the odds of DDs in model 1 (OR: 1.92; 95% CI: 1.11–3.33), but not in model 2 (OR: 1.65; 95% CI: 0.89–3.06) and model 3 (OR: 1.34; 95% CI: 0.44–4.04).

As presented in Table 2, when divided into quartiles, HbAA was not associated with the odds of DDs in a linear trend in model 1 (*P* for trend 0.116), model 2 (*P* for trend 0.286), and model 3 (*P* for trend 0.789). Similarly, HbGA was not associated with the odds of DDs in a linear trend in model 1 (*P* for trend 0.065), model 2 (*P* for trend 0.278), and model 3 (*P* for trend 0.783). The associations between HbAA or HbGA and the odds of DDs remained consistent in different subgroups by age, gender, and race (Table 3).

**Table 3:** Interaction analyses of the associations of HbAA and HbGA with developmental disabilities^#^.

	**Quartile 1**	**Quartile 2**	**Quartile 3**	**Quartile 4**	P for interaction*
**HbAA (pmol/g Hb)**	
Age, 6–12 years	Reference	0.82 (0.29–2.36)	1.79 (0.59–5.43)	0.92 (0.35–2.45)	0.948
Age, 13–17 years	Reference	1.30 (0.21–8.19)	1.27 (0.32–5.02)	1.00 (0.24–4.23)	
Male	Reference	0.72 (0.22–2.35)	1.70 (0.66–4.39)	0.92 (0.34–2.45)	0.572
Female	Reference	1.50 (0.30–7.47)	1.07 (0.19–5.93)	0.97 (0.22–4.29)	
Whites	Reference	0.86 (0.27–2.77)	1.51 (0.27–8.31)	1.15 (0.29–4.60)	0.464
Non-whites	Reference	1.41 (0.21–9.38)	2.38 (0.52–10.9)	1.23 (0.24–6.28)	
**HbGA (pmol/g Hb)**	
Age, 6–12 years	Reference	1.99 (0.86–4.58)	0.57 (0.20–1.62)	1.65 (0.68–4.01)	0.792
Age, 13–17 years	Reference	0.39 (0.10–1.51)	**0.09 (0.01–0.81)**	2.02 (0.49–8.34)	
Gender, male	Reference	1.05 (0.38–2.92)	0.36 (0.10–1.28)	1.45 (0.48–4.36)	0.437
Gender, female	Reference	1.04 (0.31–3.49)	0.20 (0.04–1.06)	1.18 (0.29–4.79)	
Race, whites	Reference	1.61 (0.52–5.02)	0.45 (0.10–1.98)	0.73 (0.24–2.25)	0.475
Race, non-whites	Reference	1.20 (0.21–6.95)	0.23 (0.03–1.65)	2.76 (0.52–14.7)	

Bold fonts mean statistically significant.

Hb, hemoglobin; HbAA, hemoglobin adducts of acrylamide; HbGA, hemoglobin adducts of glycidamide.

^#^The associations were calculated with model 3. Age, gender, race, income, body mass index, normal birth weight, maternal smoking during pregnancy, health insurance coverage, and the number of annual health care visits were adjusted in model 3.

^*^P-values were calculated by adding an interaction term between HbAA or HbGA and subgroup status in the regression model.

**Figure 1:**
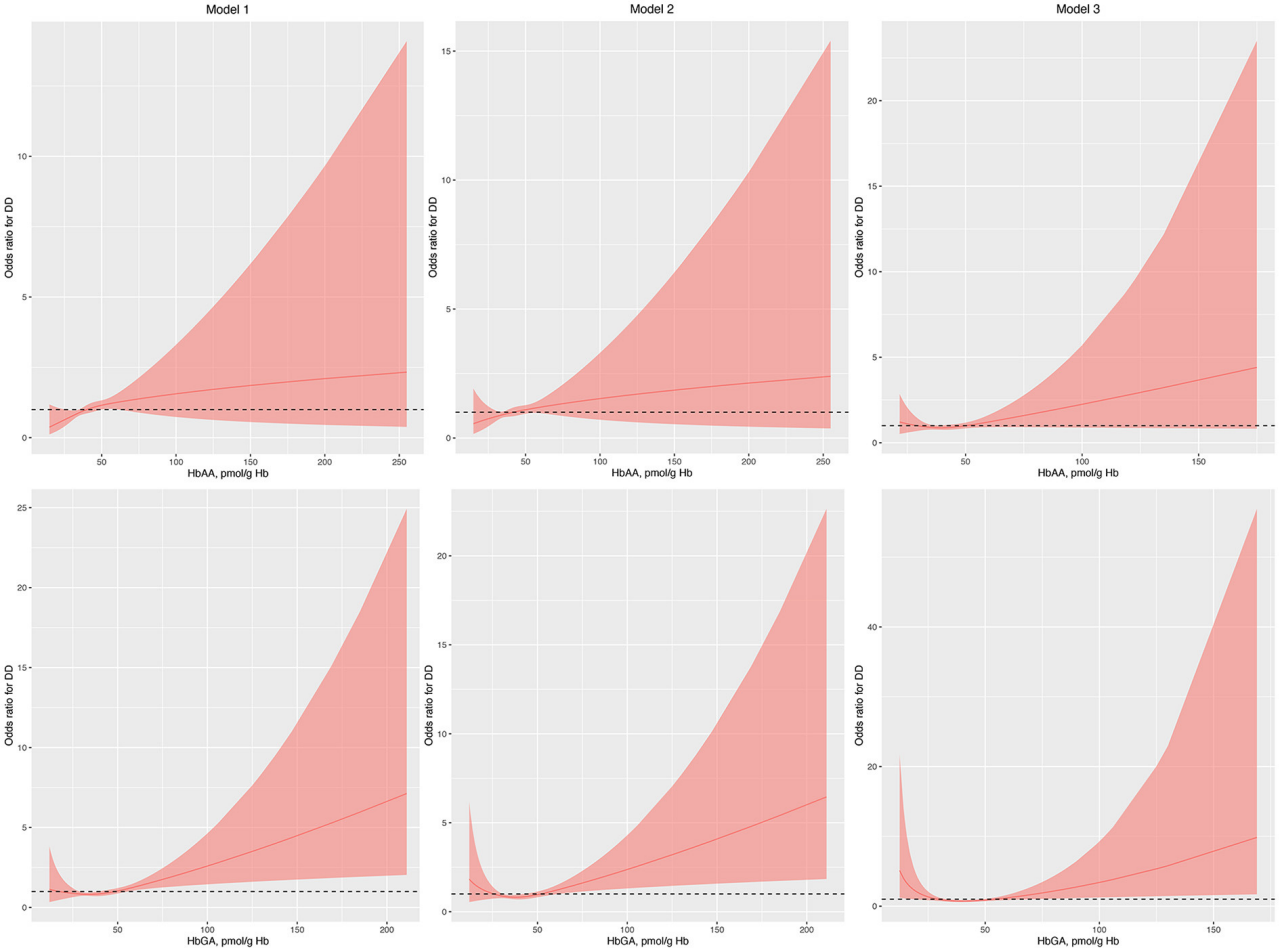
Odds ratio (95% confidence interval) of developmental disabilities by blood HbAA and HbGA levels. Odds ratio (solid lines) and 95% confidence interval (curved lines) were based on binomial regression analysis with restricted cubic splines for log-transformed HbAA and HbGA levels. No covariables were adjusted for in model 1. Age, gender, race, and income were adjusted in model 2. Age, gender, race, income, BMI, normal birth weight, maternal smoking during pregnancy, health insurance coverage, and the number of annual health care visits were adjusted in model 3. BMI, body mass index; DDs, developmental disabilities; Hb, hemoglobin; HbAA, hemoglobin adducts of acrylamide; HbGA, hemoglobin adducts of glycidamide.

Regression analysis with RCS showed that HbAA was not associated with the odds of DDs in model 1, model 2, and model 3 (Figure 1). Conversely, HbGA was significantly associated with the odds of DDs in model 1, model 2, and model 3, and there was a J-shaped association between them in model 3 (*P* for non-linearity, 0.023; Figure 1).

**Figure 2:**
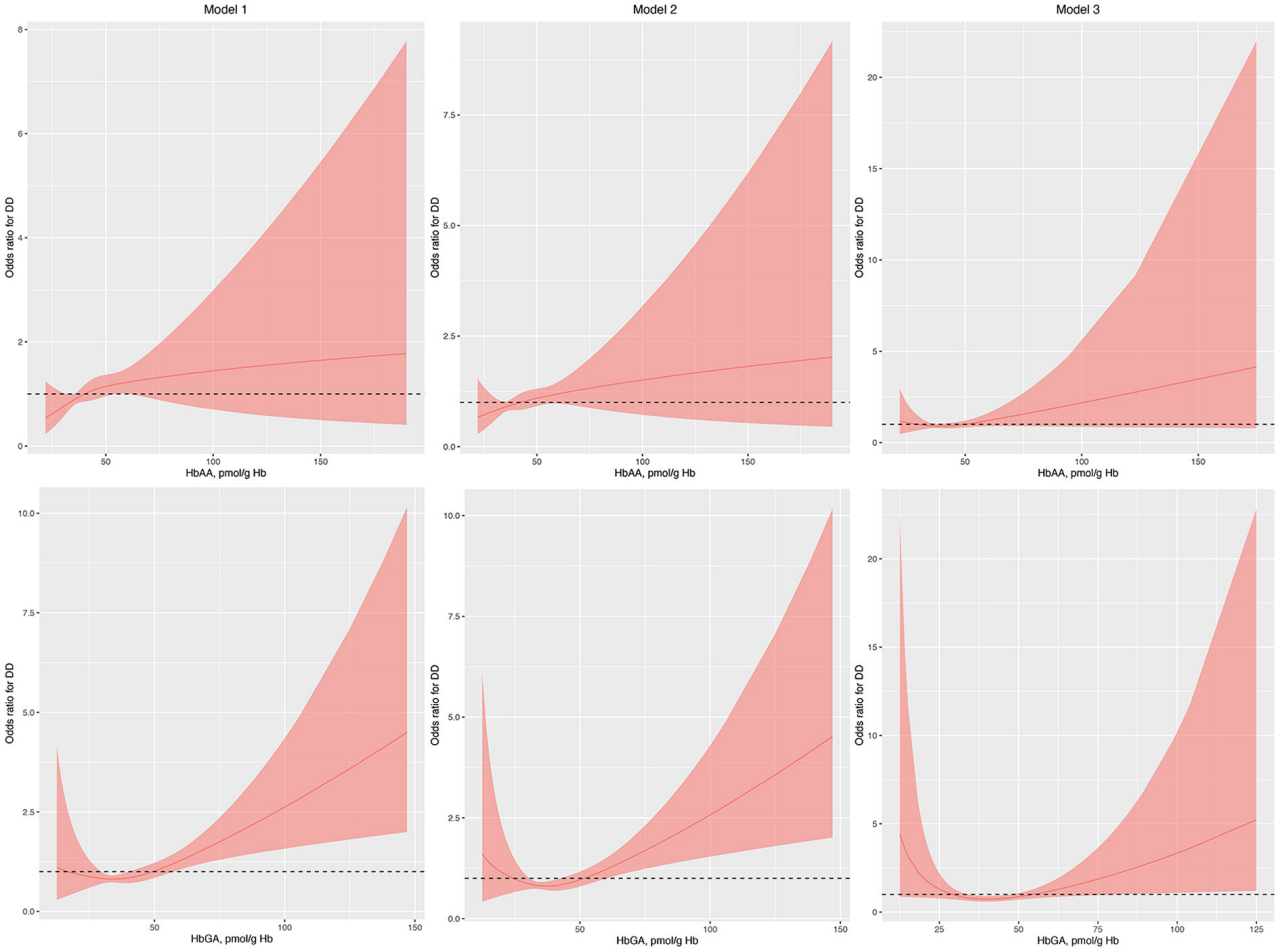
Odds ratio (95% confidence interval) of developmental disabilities by blood HbAA and HbGA levels after propensity score matching. Odds ratio (solid lines) and 95% confidence interval (curved lines) were based on binomial regression analysis with restricted cubic splines for log-transformed HbAA and HbGA levels. No covariables were adjusted for in model 1. Age, gender, race, and income were adjusted in model 2. Age, gender, race, income, BMI, normal birth weight, maternal smoking during pregnancy, health insurance coverage, and the number of annual health care visits were adjusted in model 3. BMI, body mass index; DDs, developmental disabilities; Hb, hemoglobin; HbAA, hemoglobin adducts of acrylamide; HbGA, hemoglobin adducts of glycidamide.

Gender, race, normal birth weight, and health insurance coverage were used to perform PSM as they differed between children with and without DDs. After PSM, the characteristics of children with and without DDs were similar (Supplementary Table S1). The associations between HbAA and the odds of DDs remained insignificant in model 1, model 2, and model 3 in the regression analysis with RCS (Figure 2). Conversely, the associations between HbGA and the odds of DDs remained significant in model 1, model 2, and model 3 (Figure 2).

## Discussion

In this national representative cross-sectional study, we found that the level of HbGA was associated with the odds of parent-reported DDs in a J-shaped manner in US children aged 6–17 years old even after adjustment for the potential confounders. The association was similar in different subgroups by age, gender, and race. However, we did not detect a significant association between the level of HbAA and the odds of DDs.

As a large-scale epidemiological survey conducted in the US, NHANES provides a unique opportunity to explore the role of exposure to acrylamide in health and disease among the general population. For example, Vesper et al. described human exposure to acrylamide in the general US population through the measurement of HbAA and HbGA. They found that age, gender, and race/ethnicity do not strongly affect acrylamide exposure. Smokers had the highest levels of HbAA and HbGA, and tobacco smoke exposure in nonsmokers had a significant effect on HbAA and HbGA levels (29). This result was consistent with our finding that current household tobacco smoke exposure was significantly associated with levels of HbAA and HbGA among children. Moreover, based on the data from NHANES, exposure to acrylamide was found to be associated with the risk of cardiovascular disease as well as the all-cause and cardiovascular disease mortality (15, 30).

To the best of our knowledge, this is the first study that investigates the association of acrylamide with the odds of DDs in children. Acrylamide exposure can lead to neurological symptoms in humans and numerous cases of acrylamide poisoning after occupational exposure have been reported (31, 32). For example, tunnel workers exposed to grouting agents containing acrylamide resulted in mild and reversible peripheral nervous system symptoms (32). The level of HbAA was shown to correlate with neurologic symptoms in this study, and participants with levels >1 nmol/g Hb experienced tingling or numbness in hands or feet. However, only two studies reported the possible association between dietary acrylamide exposure and neurological symptoms among the general population (18, 19). The reported symptoms included hearing loss, mild cognitive decline, and an increased risk of poor cognition. Particular concerns have been expressed over the impact of acrylamide on brain development and possible neurodevelopmental disorders. Whereas no studies have reported the effects of prenatal exposure to acrylamide on neurodevelopment (33), several cohort studies demonstrated that maternal dietary acrylamide intake was associated with reduced fetal growth including birth head circumference (34–37). It has been proposed that the decrease in cord plasma insulin levels might be a mechanism by which dietary acrylamide exposure is associated with reduced fetal growth (38). Based on the clear evidence that acrylamide exposure can impair fetal growth, further research is needed to examine whether pre or perinatal acrylamide exposure can lead to neurodevelopmental disorders in children.

Animal studies investigating the neurodevelopmental toxicity of acrylamide have been widely conducted (33). The rats born by mothers exposed to acrylamide and fed with milk from lactating females exposed to acrylamide presented serious structural changes in the cerebral cortex. It was featured by massive increases of pyknotic neuronal cells separated by widened spaces, increased number of apoptotic cells, and increased death of Purkinje cells and granular neuronal cells (39). The vitromodels also showed that acrylamide exposure altered neuronal migration, which is a critical part of neurodevelopment (40). The underlying mechanisms of neurodevelopmental toxicity of acrylamide have been proposed (33). Acrylamide or glycidamide may interact with thiol/thiolate and seleno groups on proteins and enzymes central for neurodevelopmental function. These interactions usually result in oxidative stress, mitochondrial dysfunction, inhibited axonal transport, transcriptomic alterations, and impaired neurotransmission. Brain-derived neurotrophic factor (BDNF) and cAMP response element-binding protein (CREB) signaling have also interacted with acrylamide and alteration of these mechanisms ultimately induce learning or memory impairment and disturbed motor coordination.

The results of the current study should be explained. First, in the regression analysis with RCS in model 1, we found that for the level of HbGA “the higher, the worse.” However, after adjusting for potential covariables, a J-shaped curve was detected between the level of HbGA and the odds of DDs, suggesting some other confounders might mediate the relationship between them. In the cancer study, it has been suggested that a linear extrapolation might overestimate the actual risk for acrylamide exposure and a non-linear dose-response relationship was more appropriate for acrylamide carcinogenicity in the low-dose region. Several factors including detoxication reactions, cell cycle arrest, DNA repair, apoptosis, and immune surveillance might contribute to this non-linear relationship (41). However, future studies are needed to investigate the potential reasons leading to the J-shaped relationship between HbGA and the odds of DDs. Second, we did not find a significant relationship between HbAA and the odds of DDs. HbAA and HbGA are different compounds and might exert different effects. It might also be due to the relatively small number of participants. Actually, the relationship between HbGA and the odds of DDs also became insignificant with decreased number of children in the interaction analyses or after PSM if the ratio was 1:1 or 1:2. Further studies with an increasing number of children might be warranted.

There are several limitations of this study. First, in NHANES, children with various conditions, including autism spectrum disorder, attention deficit hyperactivity disorder, developmental delay, or speech/language impairment, are eligible to receive special education or early intervention services. Thus, the definition of DDs is broad and encompasses children with different conditions. It limited our ability to examine associations with specific causes of DDs. Second, we did not adjust for household tobacco smoke exposure in the regression analysis due to the major multicollinearity. However, the possible confounding effect of tobacco exposure might affect our results due to the numerous other harmful substances from smoke. We tried to fix this problem by limiting the participants without tobacco exposure in the subgroup analysis. Yet the number of participants was too small to reach any statistically significant results (24 with DDs and 131 without DDs). Notwithstanding, the effect size was similar after limiting the participants without tobacco exposure and our final results remained consistent when including tobacco exposure as a covariable without considering the multicollinearity in the regression model. Third, this is a cross-sectional study and any causal inferences of acrylamide on DDs could not be made. Fourth, the diagnosis of DDs was based on questionnaire data and there might be some recall bias. Nonetheless, this study has several strengths, including the reliability of NHANES data and adjustment for important covariables. In addition, the nationally representative data allows us to generalize our findings to a broader population.

In this study targeting nationally representative, multi-racial/ethnic children, we found that HbGA level was associated with the odds of DDs in a J-shaped manner. Further investigation is warranted to determine the causality and underlying mechanisms.

## Data availability statement

Publicly available datasets were analyzed in this study. This data can be found here: https://www.cdc.gov/nchs/nhanes/index.htm.

## Ethics statement

The studies involving human participants were reviewed and approved by National Center for Health Statistics (NCHS) at the Centers for Disease Control and Prevention. Written informed consent to participate in this study was provided by the participants' legal guardian/next of kin.

## Author contributions

FM designed the study. YQ collected the data and performed the statistical analysis. YW and FH searched the literature and prepared the manuscript. All authors have reviewed the results and approved the submission of the manuscript.

## Conflict of interest

The authors declare that the research was conducted in the absence of any commercial or financial relationships that could be construed as a potential conflict of interest.

## Publisher's note

All claims expressed in this article are solely those of the authors and do not necessarily represent those of their affiliated organizations, or those of the publisher, the editors and the reviewers. Any product that may be evaluated in this article, or claim that may be made by its manufacturer, is not guaranteed or endorsed by the publisher.

## Abbreviations

BMI: body mass index
CI: confidence interval
Hb: hemoglobin
HbAA: hemoglobin adducts of acrylamide
HbGA: hemoglobin adducts of glycidamide
NCHS: national center for health statistics
NHANES: national health and nutrition examination survey
OR: odds ratio
PSM: propensity score matching
RCS: restricted cubic splines.
