# Supplementary material for: Association between acrylamide exposure and the odds of developmental disabilities in children: A cross-sectional study
Source: Front Public Health. 2022 Sep 30;10:972368. doi: 10.3389/fpubh.2022.972368 (PMC9561965; doi:10.3389/fpubh.2022.972368)
Supplement: Supplementary file 1 [file Table_1.docx]

| Table S1. Characteristics of children after propensity score matching | | | |
| --- | --- | --- | --- |
| Characteristic | With DDs  (N=134) | Without DDs (N=402) | P value |
| Age, years | 11.2 (0.3) | 11.9 (0.2) | 0.095 |
| Male, % | 69.4 (4.5) | 67.1 (3.0) | 0.692 |
| Race/ethnicity, % |  |  | 0.869 |
| Hispanic | 16.0 (3.1) | 16.7 (3.4) |  |
| Non-Hispanic White | 61.7 (4.6) | 60.9 (5.0) |  |
| Non-Hispanic Black | 17.9 (3.7) | 16.5 (2.9) |  |
| Other race | 4.4 (1.1) | 6.0 (1.6) |  |
| Income-to-poverty ratio ≤ 1.3, % | 37.4 (5.7) | 29.4 (2.8) | 0.188 |
| BMI | 21.2 (0.5) | 20.9 (0.3) | 0.682 |
| Birth weight > 5.5 lb, % | 76.6 (5.0) | 83.6 (3.0) | 0.239 |
| Maternal smoking during pregnancy, % | 19.3 (5.9) | 14.0 (3.6) | 0.385 |
| With health insurance coverage, % | 98.1 (1.0) | 97.8 (0.8) | 0.796 |
| Times received healthcare ≤ 1, % | 33.8 (4.8) | 40.7 (2.3) | 0.259 |
| BMI: body mass index; DDs: developmental disabilities | | | |
